# New insights on iron overload-mediated lysosomal membrane rupture in liver regeneration following acetaminophen-induced liver injury

**DOI:** 10.37349/edd.2026.1005138

**Published:** 2026-09-28

**Authors:** Hualong Zhu, Peng Cao, Wen-Xing Ding

**Affiliations:** 1 Department of Pharmacology, Toxicology and Therapeutics, University of Kansas Medical Center, Kansas City, KS 66160, USA; 2 Department of Internal Medicine, Division of Gastroenterology, Hepatology & Mobility, University of Kansas Medical Center, Kansas City, KS 66160, USA

**Keywords:** autophagy, ferroptosis, iron, liver regeneration, STEAP4

## Abstract

Acetaminophen (APAP) overdose is a leading cause of liver failure in Western countries and a major clinical challenge because effective treatment options remain limited for patients who present after the optimal early therapeutic window. Iron is distributed among several subcellular compartments, such as cytosol, mitochondria, ferritin, and endosomes/lysosomes. Emerging evidence indicates that intracellular iron homeostasis and organelle function may be critical during the regeneration phase for repairing liver damage. Our recent work identified the metalloreductase six-transmembrane epithelial antigen of the prostate 4 (STEAP4) as a critical regulator of lysosomal iron homeostasis and membrane integrity during the regeneration phase following APAP overdose. Hepatic STEAP4 deficiency promoted lysosomal iron accumulation and impaired hepatocyte proliferation, whereas deferiprone-mediated iron chelation restored lysosomal function and promoted liver regeneration. In this perspective, we discuss the potential of targeting lysosomal iron to promote liver repair after APAP overdose-induced liver injury, as well as the limitations and challenges.

## Introduction

Acetaminophen-induced liver injury (AILI) is a significant clinical challenge, particularly for patients presenting beyond the early therapeutic window, when effective strategies to promote late-phase liver regeneration remain limited [1, 2].

Intracellular iron is organized into dynamic, specialized compartments to enable the utilization, storage, and safe handling of this reactive metal [3]. The primary compartments include the cytosolic labile iron pool (LIP), mitochondria (heme and Fe-S cluster synthesis), ferritin (storage), endosomes, and lysosomes [4]. The cytosolic LIP provides a readily available source of iron for enzymatic reactions and biosynthetic processes [5]. Mitochondria use iron for heme synthesis, iron-sulfur cluster biogenesis, oxidative phosphorylation, and numerous redox reactions [3]. Ferritin stores excess iron in a relatively safe form, thereby preventing uncontrolled redox cycling [6]. Endosomes and lysosomes regulate iron uptake, recycling, storage, and redistribution through transferrin receptor (TFR)-mediated endocytosis, ferritinophagy, and degradation of iron-containing organelles [4, 7]. The compartmentalization of intracellular iron is essential for maintaining iron homeostasis and preventing iron toxicity. Thus, iron homeostasis is not simply a matter of total cellular iron content. The subcellular localization of iron is equally important, as iron overload in vulnerable compartments may also trigger organelle dysfunction.

Previous clinical and experimental studies have implicated hepatic iron redistribution, altered hepcidin responses, and iron-dependent oxidative reactions in acetaminophen (APAP) hepatotoxicity [8–10]. In the liver, hepatocytes take up ferric iron (Fe^3+^)-transferrin bound to TFRs via receptor-mediated endocytosis. The Fe^3+^-transferrin accumulates in endosomes and lysosomes, where Fe^3+^ is reduced to ferrous iron (Fe^2+^), released into the cytosol, and taken up by mitochondria to support mitochondrial biosynthesis of heme and non-heme iron-sulfur clusters. Following an APAP overdose, the APAP reactive metabolite may permeabilize lysosomes, leading to excessive iron release and mitochondrial uptake of Fe^2+^ via the mitochondrial calcium uniporter. Increased mitochondrial Fe^2+^ promotes the formation of the highly reactive hydroxyl radical (•OH) via the Fenton reaction, leading to mitochondrial lipid peroxidation, the onset of mitochondrial permeability transition, and hepatocyte death [11]. These studies established an important role of intracellular compartment iron dysregulation in AILI, primarily during the early injury phase.

In a recent Hepatology study [12], we provide novel insights into the regulation of organelle-specific iron homeostasis in the late liver regeneration instead of the focus on early necrotic events. We identified the metalloreductase six-transmembrane epithelial antigen of the prostate 4 (STEAP4) as a critical regulator of lysosomal iron balance and membrane integrity, which are critical for supporting hepatocyte proliferation after AILI.

### STEAP family protein and endosome and lysosome iron

STEAP family proteins, including STEAP1/2/3/4, are metalloreductases that reduce Fe^3+^ to Fe^2+^, cupric ion (Cu^2+^) to Cu^+^, and are essential for efficient iron and copper transport across biological membranes [13]. In particular, this function is important for endosomal and lysosomal iron transport. In this process, ironbound transferrin binds to TFR and enters endosomes and then lysosomes via endocytosis, where acidification promotes the release of Fe^3+^ from transferrin [14]. Fe^3+^ must first be reduced to Fe^2+^ before being transported into the cytosol via divalent metal transporters such as divalent metal transporter 1 (DMT1) [15, 16]. Therefore, STEAP family proteins serve as key hubs for transporting iron from endosomes and lysosomes to the cytosol by reducing Fe^3+^ to Fe^2+^. Therefore, endosomes and lysosomes are important for intracellular iron recycling, as they not only mediate transferrin uptake but also promote ferritinophagy, mitochondrial degradation, and heme protein turnover [17]. Under physiological conditions, endosomes and lysosomes facilitate iron redistribution to the cytosol, mitochondria, and iron storage pools. The low-pH endosomal and lysosomal environment promotes the release of iron from degraded substrates. Excess Fe^2+^ participates in Fenton reactions, generating reactive radicals that induce lipid peroxidation, ultimately leading to ferroptosis [18]. However, under stress conditions, excessive accumulation of lysosomal iron may become toxic. Lysosomal iron accumulation may increase susceptibility to ferroptosis by sustaining lysosome lipid peroxidation, while lysosomal membrane permeabilization (LMP) may further release iron into the cytosol, expand the LIP, and amplify ferroptosis [19, 20].

Among the STEAP family proteins, STEAP4, a protein highly expressed in metabolic tissues and immune cells, is associated with not only iron but also copper homeostasis [21–23]. Importantly, publicly available Genotype-Tissue Expression (GTEx) and Human Protein Atlas datasets confirm STEAP4 expression in normal human liver, but its role in liver biology remains largely elusive.

### STEAP4 and endosomal and lysosomal iron accumulation in AILI

In a recent study, we investigated the role of hepatic STEAP4 in regulating lysosomal iron accumulation and lysosomal membrane integrity in hepatocyte proliferation in the context of APAP overdose. First, we showed that the expression of STEAP4 decreased in the livers of humans with APAP overdose and mice. Second, it is well known that APAP induces liver injury through glutathione (GSH) depletion, c-Jun N-terminal kinase (JNK) activation, mitochondrial dysfunction, and hepatocellular necrosis. However, liver-specific STEAP4 knockout mice showed no effect on hepatic APAP protein adduct formation, GSH depletion, JNK activation, or serum alanine aminotransferase (ALT) elevation after APAP treatment for 6 and 24 hours. Third, STEAP4 knockout mice exhibited elevated serum ALT levels, increased necrotic areas, enhanced hepatic iron deposition, reduced mechanistic target of rapamycin (mTOR) activity, impaired mitophagy, and decreased hepatocyte proliferation after 48 hours of APAP treatment. These data suggest that STEAP4 is not essential for APAP metabolism and APAP-induced hepatocyte death in the early injury phase but is critical for liver repair and regeneration in the late phase of APAP overdose. Mechanistically, STEAP4 deficiency further increased APAP-induced FerroOrange-positive iron signals, which markedly colocalized with the lysosomal marker lysosome-associated membrane protein 1 (LAMP1). This suggests that STEAP4 deficiency promotes lysosomal iron accumulation in APAP-treated mice. Meanwhile, increased Galectin-3 puncta and cytosolic cathepsin B in APAP-treated STEAP4 KO hepatocytes indicated lysosomal membrane damage. Therefore, STEAP4 deficiency promotes lysosomal iron accumulation, which damages lysosomal membranes and function, leading to impaired hepatocyte proliferation.

Increased cellular iron levels can lead to ferroptosis, which is associated with glutathione peroxidase 4 (GPX4) dysfunction and increased lipid peroxidation. Because STEAP4 also reduces Cu^2+^ to Cu^+^ to mediate copper transport, it is possible that STEAP4 may also play a role in cuproptosis, a newly defined programmed cell death in which Cu^+^ accumulation triggers the aggregation of mitochondrial lipoylated proteins [24]. A recent study showed that Li exposure is associated with miscarriage, likely due to Li-induced forkhead box O1 (FOXO1)-mediated STEAP4 upregulation and placental cuproptosis [21]. Because loss of hepatic STEAP4 did not affect APAP-induced hepatocyte death, STEAP4 is less likely to be critical in either ferroptosis or cuproptosis in AILI. Nonetheless, future work is needed to better elucidate the roles of ferroptosis and cuproptosis by using a genetic approach to inhibit specific proteins in these pathways in APAP-treated mice.

It should also be noted that other STEAP family proteins in the liver may compensate for the loss of STEAP4, complicating the interpretation of the observations. However, based on the Protein Atlas, STEAP1 is mainly expressed in the brain and prostate and is undetectable in the liver. STEAP2 is primarily expressed in the brain and breast. However, STEAP3 is highly expressed in the liver and bone marrow. Therefore, STEAP3 may compensate for the loss of STEAP4 in regulating hepatic iron and copper transport and homeostasis. Since FerroOrange selectively detects Fe^2+^, which increased in the lysosomes of APAP-treated STEAP4 knockout hepatocytes, these data suggest that other STEAP family members, likely STEAP3, may help reduce Fe^3+^ to Fe^2+^ in the absence of STEAP4. The accumulation of Fe^2+^ in the lysosomes could be due to defects in Fe^2+^ export from lysosomes in the absence of STEAP4. It also remains unclear whether DMT1 would directly interact with STEAP4 and whether loss of STEAP4 would lead to decreased DMT1, resulting in lysosomal retention of Fe^2+^. In the future, it will be helpful to generate double STEAP3 and STEAP4 knockout mice to better understand hepatic iron and copper trafficking and homeostasis, as well as cell death and proliferation in AILI.

Another major finding in the study was that post-treatment with the iron chelator deferiprone (DFP, 20 mg/kg, i.p., administered 2 or 6 hours after APAP) restored lysosomal function and promoted hepatocyte proliferation, suggesting that targeting iron homeostasis may facilitate liver repair and regeneration following AILI. This is particularly important because many patients with APAP overdose have missed the early therapeutic window for *N*-acetylcysteine (NAC) treatment. Unlike NAC, which targets early metabolic injury, iron chelation may promote late-stage repair by maintaining lysosomal function. However, DFP is a systemic iron chelator with well-known side effects, including agranulocytosis, neutropenia, and systemic iron depletion, which could be harmful in a context where adequate iron is needed for hepatocyte proliferation. Therefore, the safety of using DFP to treat patients with APAP overdose needs further evaluation. Furthermore, the efficacy, optimal dose, and therapeutic window of DFP in patients with APAP overdose remain unknown. Future studies could focus on developing targeted interventions to precisely modulate lysosomal iron homeostasis, such as lysosome-specific iron chelators delivered via targeted delivery systems, to locally alleviate lysosomal oxidative stress without disrupting systemic iron metabolism.

### Possible roles and mechanisms of iron-mediated lysosomal membrane rupture and repair

LMP refers to loss of membrane integrity that permits leakage of lysosomal contents, whereas lysosomal rupture represents more extensive membrane disruption [25]. Limited membrane damage may be repaired, whereas severe damage may progress to rupture [26]. Damaged lysosomes increase calcium ion (Ca^2+^) efflux, which acts as a crucial signal for recruiting membrane repair factors, including endosomal sorting complex required for transport (ESCRT) machinery [27], phosphatidylinositol 4-kinase type 2 alpha (PI4K2A) [28], lipid transfer proteins, autophagy-related protein 2 (ATG2), oxysterol-binding protein-related protein 9/10/11 (ORP9/10/11), oxysterol-binding protein (OSBP) and ORP1L [29] or conjugation of ATG8 proteins to single membranes, also referred to as non-canonical autophagy or membrane ATG8ylation [30]. If the damage is too severe to repair, damaged lysosomes can be removed by autophagy via a selective process called lysophagy [31]. It is highly likely that both permeabilization and rupture of the lysosomal membrane may occur, with heterogeneous lysosomes even in the same cell following APAP overdose. However, whether lysosomal repair and lysophagy would play a role in AILI has not been investigated.

Multiple mechanisms may explain how lysosomal iron accumulation promotes membrane damage. First, lysosomes facilitate iron-driven oxidative reactions. The low-pH lysosomal environment promotes iron release from degraded proteins and organelles [32]. Subsequently, accumulated Fe^2+^ can generate reactive radicals through intralysosomal Fenton reactions, which attack lipids and proteins in the lysosomal membrane, thereby causing lipid peroxidation, reduced membrane stability, and lysosomal membrane rupture [33, 34]. Secondly, *N*-acetyl-*p*-benzoquinone imine (NAPQI), the highly reactive metabolite of APAP, may also permeabilize lysosomes, promoting iron release and mitochondrial uptake of Fe^2+^ via the mitochondrial calcium uniporter, thereby increasing mitochondrial reactive oxygen species (ROS) production, triggering the mitochondrial permeability transition, and leading to cell death [11, 35]. Third, Granzyme B treatment increased mitochondrial ROS production, which is necessary to induce lysosomal membrane rupture in U937 cells [36]. Therefore, APAP-induced mitochondrial damage may also exacerbate lysosomal membrane rupture, although it has not been investigated directly. On the other hand, earlier studies show that the release of lysosomal cathepsins, such as cathepsin B, can also induce mitochondrial damage, thereby promoting tumor necrosis factor alpha (TNF-α)-induced apoptosis in hepatocytes [37, 38]. Finally, lysosomes are the terminal stage of autophagic degradation; thus, lysosomal damage also impairs autophagic degradation, including mitophagy [39, 40]. Therefore, it is tempting to hypothesize that iron overload-mediated lysosomal dysfunction impairs mitophagy and mitochondrial dysfunction, leading to the accumulation of damaged mitochondria and increased oxidative stress, thereby further promoting lysosomal membrane rupture in a vicious cycle. In addition to lysosomes, STEAP4 has been reported to localize to early endosome antigen 1 (EEA1)-positive early endosomes, a finding we also confirmed in primary cultured mouse hepatocytes [12, 41]. Whether STEAP4 would also affect iron homeostasis in early endosomes, and whether and how it would affect liver regeneration in APAP-treated liver-specific STEAP4 knockout mice, remains unclear.

Previous studies have established that lysosomal membrane integrity and lysosomal degradation capacity are essential for nutrient sensing and for recruiting mTOR to the lysosomal membrane to activate it. Increased lysosomal amino acids from protein degradation promote Ragulator activation, which further recruits mTOR to the lysosomal surface for activation. Because loss of STEAP4 impaired lysosomal functions, as demonstrated by reduced cathepsin activity, it is speculated that these impairments would lead to decreased amino acid levels and failure to recruit mTOR to the lysosomal surface, thereby inhibiting mTOR activation. Decreased mTOR activation further decreased the phosphorylation of S6K and 4-EBP1, thus inhibiting protein synthesis and cell proliferation in APAP-treated STEAP4 knockout mice. Furthermore, lysosomes are the terminal stage for autophagic degradation, and increased lysosomal damage can impair autophagic degradation, which is essential for cell proliferation by providing the necessary lipids and biomolecules as building blocks [42, 43]. In addition to general autophagy, decreased lysosomal function also impairs mitophagy, which may further reduce adenosine triphosphate (ATP) production and mitochondrial metabolite availability from the tricarboxylic acid (TCA) cycle, both of which are required for cell proliferation. Notably, the iron chelator DFP can reverse lysosomal damage and promote hepatocyte proliferation in APAP-treated liver-specific STEAP4 knockout mice, suggesting these adverse effects are mediated by increased iron retention. Therefore, lysosomal iron accumulation may promote AILI through multiple complementary mechanisms, including lysosomal damage, impaired mTOR signaling and mitochondrial quality control, and potentially reduced availability of biomolecule-building fuels. Nonetheless, whether lysosomal mTOR recruitment is impaired, leading to inhibition of mTOR, which acts as a causal factor for impaired mitophagy and liver regeneration in APAP-treated liver-specific STEAP4 knockout mice, remains to be studied in the future.

Transcription factor EB (TFEB) is a master regulator of gene expression for lysosomal biogenesis, which is critical for meeting the demand for autophagic degradation and for replacing and repairing damaged lysosomes. In addition to lysosomal genes, TFEB regulates gene expression for mitochondrial biogenesis by upregulating peroxisome proliferator-activated receptor gamma coactivator 1-alpha (PGC1-α) in hepatocytes and adipocytes. Therefore, overexpression of TFEB increased hepatocyte proliferation in APAP-treated mice. This is likely due to increased biogenesis of lysosomes and mitochondria, as well as increased mitophagy and lysophagy, which provide biomolecules for cell proliferation via lysosomal degradation and ATP, respectively. Indeed, pharmacological activation of TFEB increased hepatocyte proliferation and protected against AILI in mice [44, 45]. A proposed schematic model of how STEAP4 may regulate lysosomal iron homeostasis and LMP in AILI is shown in Figure 1.

### Future perspectives

While current findings delineate the pathological role of lysosomal iron dyshomeostasis and LMP in impairing APAP-induced liver regeneration, translational applications in this field warrant further investigation. DFP showed promising therapeutic effects by chelating cellular iron and promoting recovery of lysosomal function and hepatocyte proliferation. However, DFP is a systemic, non-lysosome-specific iron chelator, and excessive iron chelation may disrupt physiological iron homeostasis and cause serious adverse effects such as agranulocytosis and neutropenia. Clinical translation will therefore require careful evaluation of dose, timing, treatment duration, safety, and lysosome-targeted delivery. Future studies could focus on developing targeted interventions to specifically deliver iron chelators to hepatocytes by screening iron chelators that are transported only via hepatocyte-specific transporters such as OATPs. To precisely modulate lysosomal iron homeostasis, it is necessary to develop lysosome-specific iron chelators that locally chelate lysosomal iron and alleviate lysosomal oxidative stress without disrupting systemic iron metabolism. Since lysosomes are acidic, identifying basic iron chelators would help specifically target acidic lysosomes. Furthermore, for late-stage liver injury post APAP, where the optimal therapeutic window for NAC has closed, identifying small-molecule compounds that safely activate lysosomal repair and lysophagy pathways to stimulate hepatocyte proliferation could complement current NAC treatment. Finally, evaluating whether the aberrant iron-lysosome axis similarly contributes to the pathogenesis of other liver diseases, such as metabolic dysfunction-associated steatotic liver disease (MASLD), alcohol-associated liver disease, or hepatic ischemia-reperfusion injury, will help elucidate the broader mechanistic role of organelle-specific iron dyshomeostasis in various liver diseases and compromised tissue regeneration.

## Figures and Tables

**Figure 1.**
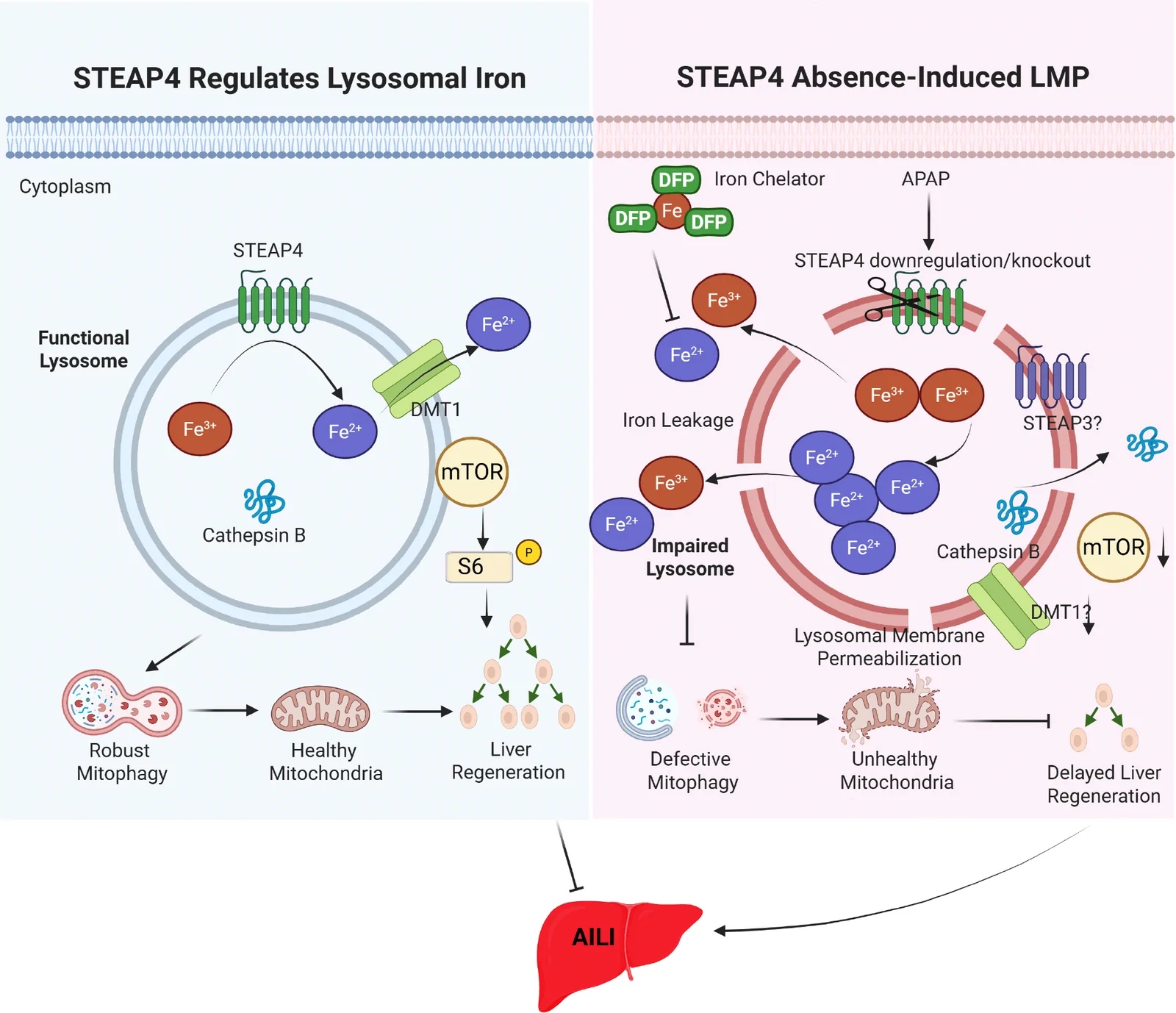
A proposed schematic model for the role of STEAP4 in regulating lysosomal iron homeostasis and dysfunction in APAP-induced liver injury. In normal hepatocytes, lysosomal STEAP4 reduces Fe^3+^ to Fe^2+^ via its metalloreductase function in the lysosomal lumen. Fe^2+^ is then transported out of lysosomes to the cytosol via DMT1. Functional lysosomes also recruit mTOR to the lysosomal surface for activation, leading to increased phosphorylation of S6K and 4-EBP1, thereby increasing protein synthesis and hepatocyte proliferation. Functional lysosomes also serve as the endpoint for autophagic degradation, including mitophagy, which removes damaged mitochondria and provides biomolecules for hepatocyte proliferation. In the absence of STEAP4 due to APAP overdose or genetic deletion of STEAP4 in hepatocytes, lysosomal Fe^2+^ levels increase, likely due to compensatory effects from STEAP3 and possible downregulation of DMT1. This may lead to lysosomal damage, with increased lysosomal membrane permeabilization and rupture, resulting in the release of iron and cathepsins from lysosomes. Damaged lysosomes may inhibit lysosomal mTOR recruitment and activation and impair mitophagy, resulting in decreased hepatocyte proliferation and impaired liver regeneration following APAP overdose. “?”: denotes an unknown event. Fe^2+^: ferrous iron; Fe^3+^: ferric iron; mTOR: mechanistic target of rapamycin; STEAP4: six-transmembrane epithelial antigen of the prostate 4.

## Data Availability

Not applicable.

## Abbreviations

•OH: hydroxyl radical
AILI: acetaminophen-induced liver injury
ALT: alanine aminotransferase
APAP: acetaminophen
ATG2: autophagy-related protein 2
ATP: adenosine triphosphate
Ca^2+^: calcium ion
Cu^2+^: cupric ion
DFP: deferiprone
DMT1: divalent metal transporter 1
EEA1: early endosome antigen 1
ESCRT: endosomal sorting complex required for transport
Fe^2+^: ferrous iron
Fe^3+^: ferric iron
FOXO1: forkhead box O1
GPX4: glutathione peroxidase 4
GSH: glutathione
GTEx: Genotype-Tissue Expression
JNK: c-Jun N-terminal kinase
LAMP1: lysosome-associated membrane protein 1
LIP: labile iron pool
LMP: lysosomal membrane permeabilization
MASLD: metabolic dysfunction-associated steatotic liver disease
mTOR: mechanistic target of rapamycin
NAC: *N*-acetylcysteine
NAPQI: *N*-acetyl-*p*-benzoquinone imine
ORP9/10/11: oxysterol-binding protein-related protein 9/10/11
OSBP: oxysterol-binding protein
PGC1-α: peroxisome proliferator-activated receptor gamma coactivator 1-alpha
PI4K2A: phosphatidylinositol 4-kinase type 2 alpha
ROS: reactive oxygen species
STEAP4: six-transmembrane epithelial antigen of the prostate 4
TCA: tricarboxylic acid
TFEB: transcription factor EB
TFR: transferrin receptor
TNF-α: tumor necrosis factor alpha
